# β-Cyclodextrin/CMK-8-Based Electrochemical Sensor for Sensitive Detection of Cu^2+^

**DOI:** 10.3390/molecules27154954

**Published:** 2022-08-04

**Authors:** Chengqi Bao, Yan Lu, Jiawei Liu, Yansha Gao, Limin Lu, Shuwu Liu

**Affiliations:** Key Laboratory of Crop Physiology, Ecology and Genetic Breeding, Ministry of Education, Key Laboratory of Chemical Utilization of Plant Resources of Nanchang, College of of Chemistry and Materials, Jiangxi Agricultural University, Nanchang 330045, China

**Keywords:** copper ion, β-cyclodextrin, mesoporous carbon, electrochemical sensor

## Abstract

In this work, β-cyclodextrin (β-CD)/mesoporous carbon (CMK-8) nanocomposite was synthesized and used as an electrochemical sensing platform for highly sensitive and selective detection of Cu^2+^. The morphology and structure of β-CD/CMK-8 were characterized by scanning electron microscope (SEM) and X-ray diffraction (XRD). In addition, the dates from electrochemical impedance spectroscopy (EIS) and Cyclic voltammetry (CV) demonstrated that the β-CD/CMK-8 possessed a fast electronic transfer rate and large effective surface area. Besides this, the β-CD/CMK-8 composite displayed high enrichment ability toward Cu^2+^. As a result of these impressive features, the β-CD/CMK-8 modified electrode provided a wide linear response ranging from 0.1 ng·L^−1^ to 1.0 mg·L^−1^ with a low detection limit of 0.3 ng·L^−1^. Furthermore, the repeatability, reproducibility and selectivity of β-CD/CMK-8 towards Cu^2+^ were commendable. The sensor could be used to detect Cu^2+^ in real samples. All in all, this work proposes a simple and sensitive method for Cu^2+^ detection, which provides a reference for the subsequent detection of HMIs.

## 1. Introduction

HMIs are considered to be a group of hazard pollutants that pose a threat to the environment [1]. With the continuing development industry, most HMIs are released into the environment, causing serious pollution. The accumulation of HMIs in the food chain has great potential to harm human health [2,3]. Copper, as a crucial trace element, occupies a significant position in a myriad of physiological functions [4,5,6]. However, excessive Cu^2+^ might lead to cancer [7], cardiovascular disorders [8] and neurodegenerative diseases [9]. Therefore, it is urgent to develop an effective and sensitive method for the trace detection of Cu^2+^ in the food industry and environmental field. So far, various techniques have been developed for detecting Cu^2+^, including graphite furnace atomic absorption spectroscopy (GF-AAS) [10], atomic absorption spectrometry (AAS) [11] and inductively coupled plasma atomic emission spectrometry (ICP-AES) [12], etc. These methods display high sensitivity. However, these techniques are relatively expensive and, in most cases, require a complex and rigorous pre-treatment of the sample to be analyzed. In contrast, the electrochemical method has aroused widespread attention because of its advantages, such as its simple operation, its quick responsiveness, low cost, and high selectivity [3,13,14,15].

As an electrochemical sensor, its performance mainly depends on the electrode material. β-cyclodextrin (β-CD) is a kind of cyclic oligosaccharide composed of seven glucopyranose units with a cylindrical structure with internal hydrophobicity and external hydrophilicity [16,17]. The hydrophobic inner cavity of β-CD can selectively bind various organic, inorganic, and biological guest molecules into its cavities to form host–guest inclusion complexes [18,19,20]. When a β-CD-modified electrode is applied to detect Cu^2+^, the wide opening cavity of β-CD could form a binuclear hydroxyl bridge with Cu^2+^ to achieve the selective adsorption of Cu^2+^ [21]. Nevertheless, poor conductivity is the intrinsic shortcoming of β-CD, which limits its wide application. In this case, combining β-CD with conductive materials has been proved to be a feasible method to improve its conductivity. Ordered mesoporous carbon (OMC) has gained much attention due to its large specific surface area, large pore volume, low cost, high stability and good conductivity [22]. OMC is usually synthesized by etching silica templates and different types of carbon materials could be produced by changing the pore structure of the mesoporous silica templates [23], such as CMK-3 and CMK-8. Among them, CMK-8 is a kind of three-dimensional (3D) cubic mesoporous carbon with a robust 3D framework [24,25]. It possesses unique structural properties, such as high surface area, highly uniform mesoscale pores and an ordered mesopore system [26,27]. However, as far as we know, there has been no relevant report on the use of β-CD/CMK-8 as electrode material for the quantification of Cu^2+^.

In view of the above considerations, herein, β-CD/CMK-8 composite was successfully developed through an ultrasonic blending method and employed as an electrochemical sensing platform for the detection of Cu^2+^. The abundant hydroxyl groups in the cavity of β-CD could form a binuclear hydroxyl bridge with Cu^2+^, so as to achieve selective detection of Cu^2+^. In addition, the excellent conductivity of CMK-8 improves the interfacial electron transfer rate. More than this, the large specific surface area and porous structure of β-CD/CMK-8 provide a great quantity of active sites for the detection of Cu^2+^, which endows the Cu^2+^ sensor with a low detection limit, wide linear range, good stability and selectivity, etc.

## 2. Results and Discussion

### 2.1. Materials Characterization

**Figure 1:**
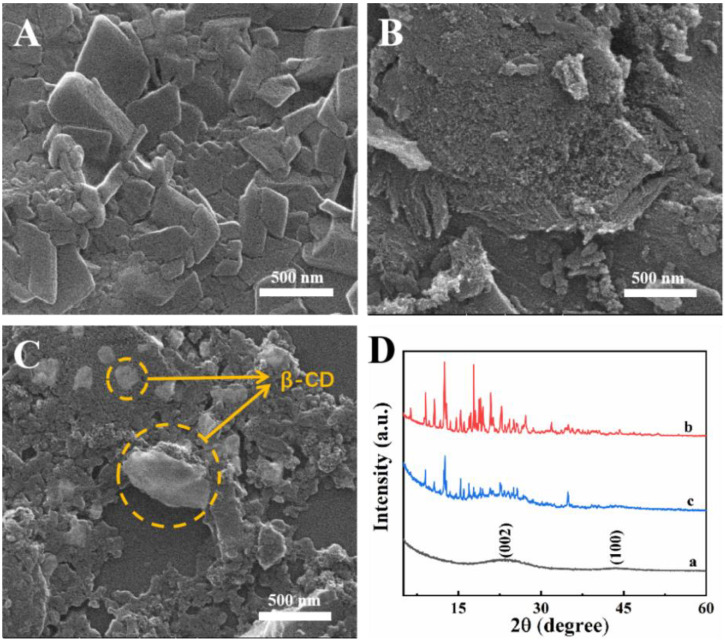
SEM images of (**A**) β-CD, (**B**) CMK-8 and (**C**) β-CD/CMK-8; (**D**) XRD patterns of CMK-8 (curve a), β-CD (curve b) and β-CD/CMK-8 (curve c).

SEM shows that the β-CD is a kind of polyhedron (Figure 1A) and CMK-8 has a rough and porous surface (Figure 1B). For β-CD/CMK-8 (Figure 1C), β-CD and CMK-8 were entangled evenly with each other, and the β-CD was exposed on the surface of CMK-8, creating more binding sites, which benefited the highly sensitive detection of Cu^2+^. The result of the SEM indicated that β-CD/CMK-8 had been synthesized successfully. Meanwhile, the crystallinity of CMK-8, β-CD and β-CD/CMK-8 were studied by XRD (Figure 1D). As shown, for CMK-8 (curve a), there were two diffraction peaks at 2θ = 27.5° and 43.0°, which were accordant with (002) and (100) crystal planes, respectively, indicating good graphitization degree. The XRD curve of β-CD (curve b) and β-CD/CMK-8 (curve c) were almost consistent, except for the slight differences at 27.5° and 43°, which indicated the characteristic peaks of β-CD and CMK-8 were retained in β-CD/CMK-8.

### 2.2. Absorption Experiments

**Figure 2:**
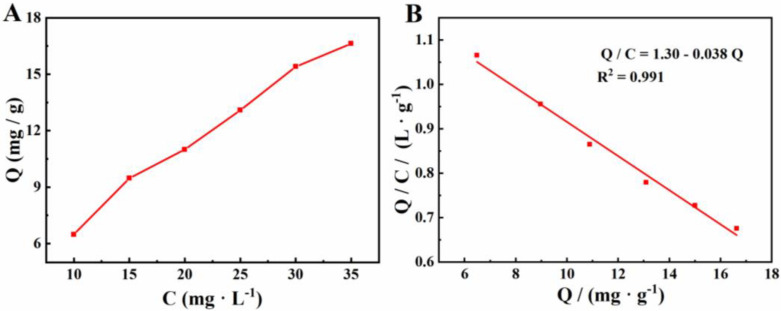
(**A**) Blinding isotherm of β-CD/CMK-8 toward Cu^2+^. (**B**) Scatchard curve of blinding nature of β-CD/CMK-8 toward Cu^2+^.

The adsorption behavior of β-CD/CMK-8 was explored by means of a static adsorption test. The adsorption isotherm is displayed in Figure 2A. The amount of Cu^2+^ adsorbed onto β-CD/CMK-8 is expressed as:

$$Q=\left(C_{0}-C\right)\;V\;/\;m$$

In which, Q (mg·g^−1^) is the adsorbed quantity of adsorbate per unit mass of the adsorbent. Concentrations of $C_{0}\;$and C (mg·L^−1^) are the initial and equilibrium of contaminants, respectively, m (g) is the mass of the adsorbent and V (mL) is the volume of adsorption solution. As shown, the Cu^2+^ adsorption capacity increased with increasing solution concentration. In addition, The maximum binding capacity ($Q_{max}$) and apparent dissociation constant ($K_{D}$) could be obtained by the Scatchard equation:

$$Q\;/\;C=\left(Q_{max}-Q\right)\;/\;K_{D}$$

The Scatchard analysis of β-CD/CMK-8 toward Cu^2+^ is presented in Figure 2B. As shown, Q has a linear relationship with Q / C , which indicated that β-CD/CMK-8 has a class of equivalent binding sites and uniform affinity for Cu^2+^. The corresponding linear regression equation is *Q/C =* 1.30 − 0.038 *Q (R^2^ =* 0.991). Thus, the maximum binding capacity ($Q_{max}$) and apparent dissociation constant ($K_{D}$) could be calculated, and were 26.32 mg·g^−1^ and 34.21 mg·L^−1^, respectively.

### 2.3. Electrochemical Characterization of Different Modified Electrodes

**Figure 3:**
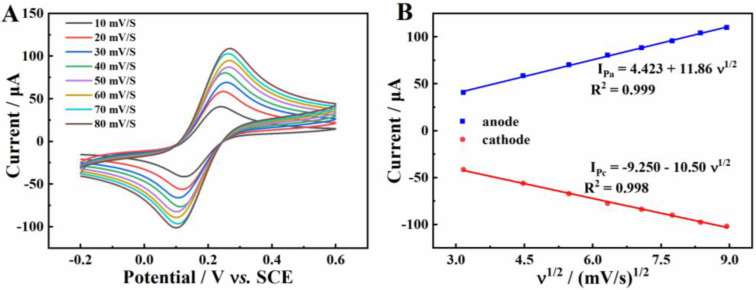
(**A**) CV curves of β-CD/CMK-8/GCE at different scan rates; (**B**) The linear relationship between the peaks current and v^1/2^.

The effective surface area ($A_{eff}$) of β-CD/CMK-8/GCE was calculated according to the Randles-Sevcik equation [28,29]. Roughness factor ($R_{f}$) is also a pivotal parameter to characterize the composite.

$$I_{p}=2.99\times 10^{5}\;n^{3/2}\;A_{eff}\;D_{0}^{1/2}\;C\;v^{1/2}$$

$$R_{f}=A\;/\;A_{geom}$$

where, $I_{p}$ refers to the maximum current of anode. $A_{eff}$ represents the effective surface area of β-CD/CMK-8/GCE. $D_{0}$ (7.6 × 10^−6^ cm^2^·s^−1^) is the diffusion coefficient. n represents the electron transfer number (n = 1). C is the concentration of the probe molecule (5 mM [Fe (CN)_6_] ^3−/4−^). v is the scan rate (0.05 V s^−1^). $A_{geom}$ is the geometric surface area (0.03925 cm^2^). Figure 3A shows CVs cureves of β-CD/CMK-8/GCE at different scan rates. According to these parameters (shown in Figure 3B), the effective surface area and roughness factor of β-CD/CMK-8 were estimated to be 0.091 cm^2^ and 2.32, respectively, which were significantly higher than those of bare GCE ($A_{eff}$ = 0.0707 cm^2^, $R_{f}$ = 1). The β-CD/CMK-8 provided a larger effective surface area for electrochemical reaction, which had great significance for enhancing the sensitivity of the sensor.

**Figure 4:**
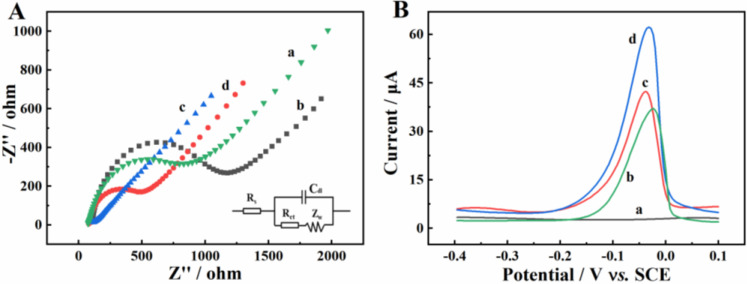
(**A**) Electrochemical impedance spectroscopy of bare GCE (a), β-CD/GCE (b), CMK-8/GCE (c) and β-CD/CMK-8 /GCE (d); (**B**) DPASV of 1.0 mg·L^−1^ Cu^2+^ at bare GCE (a), β-CD/GCE (b), CMK-8/GCE (c) and β-CD/CMK-8/GCE (d) in 0.1 M ABS (pH 5.0).

Electrochemical impedance spectroscopy (EIS) is an available tool for exploring the electron transfer behavior of different modified electrodes, which usually consists of a straight line of low-frequency and a semicircle of high-frequency. The diameter of the high-frequency semicircle represents electron transfer resistance (R_ct_) [30]. Figure 4A describes the Nyquist diagrams of the GCE (curve a), β-CD/GCE (curve b), CMK-8/GCE (curve c) and β-CD/CMK-8/GCE (curve d). In addition, the impedance data can be obtained from Randles circuit model (the illustration of Figure 4A), composed of Warburg impedance (Z_w_), electrode surface resistance (R_s_), double-layer capacitance (C_dl_) and R_ct_. Accordingly, the R_ct_ value of bare GCE was 876.4 Ω, while, β-CD/GCE presented a larger semicircle with R_ct_ at about 1067.8 Ω, which was attributed to the low conductivity of β-CD. CMK-8/GCE displayed a minuscule semicircle with a R_ct_ value of 49.6 Ω, demonstrating the high conductivity of CMK-8. The R_ct_ value of β-CD/CMK-8/GCE was 417.8 Ω, which was between that of β-CD and CMK-8, confirming that the β-CD/CMK-8 composite was successfully prepared.

### 2.4. Electrochemical Responses of Cu^2+^ on Different Electrodes

The electrochemical responses toward 1.0 mg·L^−1^ Cu^2+^ at different modified electrodes were researched by DPASV (Figure 4B). It can be clearly observed that there was no obvious oxidation peak on bare GCE (curve a), while, there were evident oxidation peaks on β-CD/GCE (curve b) and CMK-8/GCE (curve c), which were due to the good Cu^2+^-enrichment ability of β-CD and the excellent conductivity of CMK-8 [24]. Meanwhile, the largest oxidation peak current was observed on β-CD/CMK-8/GCE (curve d), which was about twice that of β-CD/GCE or CMK-8/GCE. This phenomenon could be attributed to the synergetic effects between β-CD and CMK-8. These results indicated that β-CD/CMK-8 was an ideal electrode material for fabricating a Cu^2+^ sensor.

### 2.5. Optimization of the Experimental Conditions

**Figure 5:**
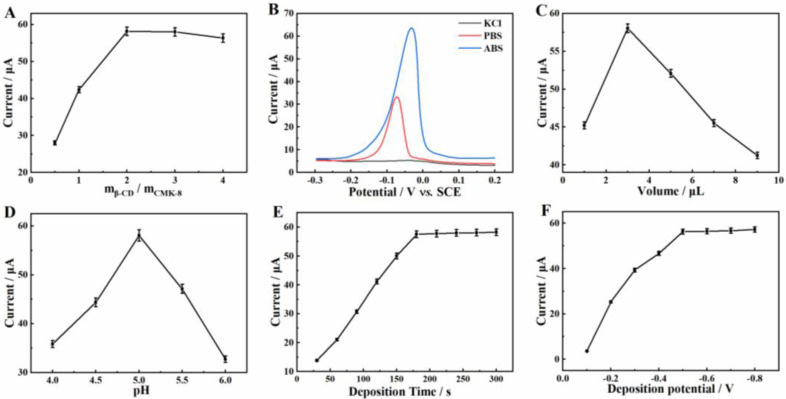
Optimization of experimental conditions. Effect of (**A**) the mass ratios of β-CD and CMK-8, (**B**) supporting electrolyte, (**C**) the volume of β-CD/CMK-8/GCE, (**D**) the buffer pH value, (**E**) the deposition time and (**F**) the deposition potential on the electrochemical response of 1 mg·L^−1^ Cu^2+^ at β-CD/CMK-8/GCE.

Some experimental parameters were optimized to explore the best conditions for the detection of Cu^2+^. The effect of the mass ratio of β-CD and CMK-8 on the response of the modified electrode to 1 mg·L^−1^ Cu^2+^ is presented in Figure 5A. As shown, the current value increased with the increase of mass ratio from 1:2 to 2:1. The result might be attributed to the fact that more active site was produced with the increase of the amount of β-CD on the surface of the electrode. However, when the mass ratio further increased to 4:1, the response current tended to be almost constant. Therefore, in this work, the mass ratio of β-CD and CMK-8 was fixed at 2:1.

The effect of the support electrolyte type was also studied. Cu^2+^ detection performances, based on β-CD/CMK-8/GCE in 0.6 M KCl solution (pH 5.0), 0.1 M phosphate buffer solution (PBS, pH 5.0) and 0.1 M HAc-NaAc buffer solution (ABS, pH 5.0) were conducted, and the results are displayed in Figure 5B. As shown, the peak current of Cu^2+^ in 0.6 M KCl solution was almost invisible (curve a), while an enhanced peak current was found in 0.1 M PBS solution (curve b). More obviously, the peak current of Cu^2+^ in 0.1 M ABS solution (curve c) was the largest. Therefore, ABS was applied as the optimal electrolyte solution for the detection of Cu^2+^.

The modification amount of β-CD/CMK-8 on GCE was optimized (Figure 5C). The result showed that the peak current of Cu^2+^ increased with the increased β-CD/CMK-8 suspension when the volume of the suspension was less than 3 μL. This could be attributed to the fact that the active site increased with the increase of modification amount. However, the peak current decreased rapidly when the volume of β-CD/CMK-8 suspension was more than 3 μL. This phenomenon was because a high amount of β-CD/CMK-8 on the electrode surface caused considerable resistance against electron transfer. Therefore, 3 μL was used as the optimal modified volume of β-CD/CMK-8 suspension.

The influence of pH value of ABS on the detection of Cu^2+^ was explored (Figure 5D). The result showed that the maximum peak current of Cu^2+^ appeared with a pH of 5.0. This might be attributed to the fact that the Cu^2+^ binding site could be protonated when the pH value was lower than 5.0, leading to the weakened adsorption of Cu^2+^. However, when the pH value was higher than 5.0, the Cu^2+^ could be hydrolyzed, resulting in a reduced response current. Therefore, 5.0 was employed as the optimum pH value.

The effect of deposition time on the detection of Cu^2+^ was explored (Figure 5E). As the deposition time increased from 30 s to 180 s, the peak current of Cu^2+^ increased gradually. The result might be attributed to the fact that with the increase of deposition time, more and more Cu^2+^ was accumulated on the surface of the electrode. However, when deposition time exceeded 180 s, the response current of Cu^2+^ tended to be flat. This phenomenon might be due to the saturation of electrode surface [1]. Thus, 180 s was selected as the optimum deposition time.

Finally, other conditions kept constant, the deposition potential from −0.1 V to −0.8 V was chosen to research the effect of deposition potentials on the response of Cu^2+^ (Figure 5F). The peak current of Cu^2+^ displayed an increasing trend as the deposition potential transferred from −0.1 V to −0.5 V, indicating that the negative shift of the deposition potential promoted the reduction of Cu^2+^ on the electrode surface. However, the peak current remained constant as the deposition potential continued to shift negatively. This might be because hydrogen evolution occurred on the electrode when the potential was too negative [31,32]. Therefore, −0.5 V was chosen as the optimum deposition potential.

### 2.6. Kinetics Studies

**Figure 6:**
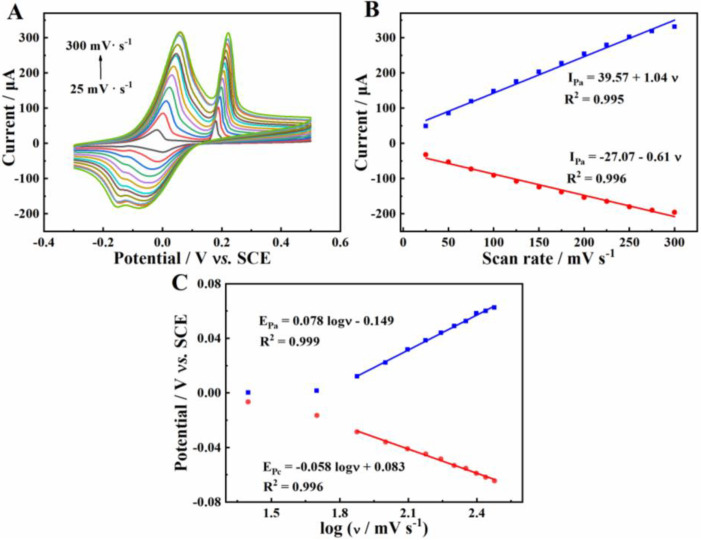
(**A**) Cyclic voltammetric response of β-CD/CMK-8/GCE to 1.0 mg·L^−1^ Cu^2+^ in ABS (pH 5.0) with scan rate of 25, 50, 75, 100, 125, 150, 175, 200, 225, 250, 275, 300 mV·s^−1^; (**B**) Relationship between the peak current and scan rate; (**C**) Relationship between peak potential and logarithm of scan rate under high scan rate.

The effect of scan rate on the redox of Cu^2+^ on β-CD/CMK-8/GCE was studied by cyclic voltammetry (CV). Figure 6A shows the CVs of 1.0 mg·L^−1^ Cu^2+^ at β-CD/CMK-8/GCE with the scan rate ranging from 25 to 300 mV·s^−1^. As shown, the redox currents increased with the increase of the scan rate. There were good linear relationships between the anodic and cathodic currents and between the scan rates and the linear regression equations of *I_pa_ =* 39.57 + 1.04 *ν (R^2^=* 0.995) and *I_pc_* = −27.07 − 0.61 *ν (R^2^ =* 0.996), respectively (Figure 6B). The phenomenon indicated that the electrochemical behavior of Cu^2+^ on β-CD/CMK-8/GCE was an adsorption control process.

Moreover, from Figure 6C, it can be seen that the anode peak potential ($E_{pa\;}$) and cathode peak potential ($E_{pc}$) had a good linear correlation with the logarithm of scan rate (log ν) under high scan rate. The linear regression equations were *E_pa_* = 0.085 *log ν −* 0.14 (*R^2^* = 0.999) and *E_pc_* = −0.059 *log ν +* 0.083 (*R^2^* = 0.996), respectively. According to the Laviron formulae, $\;E^{0\prime }$ is standard potential, the slope of the equation for $E_{pa\;}$ and $E_{pc}$ could be represented as 2.3RT / (1−α)nF and −2.3RT / αnF, respectively.

$$E_{pa\;}=E^{0\prime \;}+\left[2.3RT\;/\;\left(1-\alpha \right)nF\right]\;log\;\nu$$

$$E_{pc}=E^{0\prime \;}-\left(2.3RT\;/\;\alpha nF\right)\;log\;\nu$$

$$E_{pa\;}=E^{0\;}+\left(2.3RT\;/\;\alpha nF\right)\;log\;(RTK^{0}\;/\;\alpha +\left(2.3RT\;/\;\alpha nF\right)\;log\;\nu$$

$$log\;k_{s}=\alpha log\;\left(1-\alpha \right)+\left(1-\alpha \right)log\;\alpha -log\left(RT\;/nF\nu \right)-\alpha \left(1-\alpha \right)nF\Delta E_{P}\;/\;2.3RT$$

Consequently, electron transfer coefficient (α) and electron transfer number (n) could be calculated to be 0.59 and 1.97, respectively. Furthermore, based on the Laviron Equations $K^{0}$ and $k_{s}$ of β-CD/CMK-8/GCE were calculated to be 0.23 and 0.51 s^−1^, averaged.

### 2.7. Electrochemical Detection of Cu^2+^ at β-CD/CMK-8/GCE

**Figure 7:**
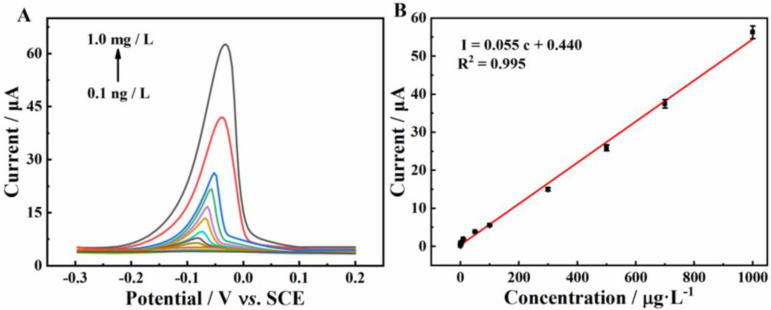
(**A**) DPASV of β-CD/CMK-8/GCE in 0.1 M ABS (pH 5.0) containing different concentrations of Cu^2+^; (**B**) The relationship between the peak current and the concentration of Cu^2+^ from 0.0001 to 1000 μg·L^−1^.

Under the optimized conditions, the electrochemical performance of Cu^2+^ on β-CD/CMK-8/GCE was researched by means of DPASV. As shown in Figure 7A, there was an obvious oxidation peak at −0.06 V, the peak current of which increased with the increase of Cu^2+^ concentration. Besides this, a good linear relationship between the concentration of Cu^2+^ and response current was established from 0.1 ng·L^−1^ to 1.0 mg·L^−1^ (R^2^ = 0.995, Figure 7B). The limit of detection (LOD) of the Cu^2+^ sensor was calculated as 0.3 ng·L^−1^ (LOD = 3 SD / S), where SD was the standard deviation of intercept and S was the sensitivity. The LOD value was far lower than the prescribed value in drinking water by the World Health Organization (2000 μg·L^−1^) [33]. The β-CD/CMK-8/GCE also possessed lower LOD and higher sensitivity than those reported in previous literature with regards to Cu^2+^ detection (shown in Table 1) [34,35,36,37,38].

### 2.8. Repeatability, Reproducibility, Stability, and Selectivity Measurements

**Figure 8:**
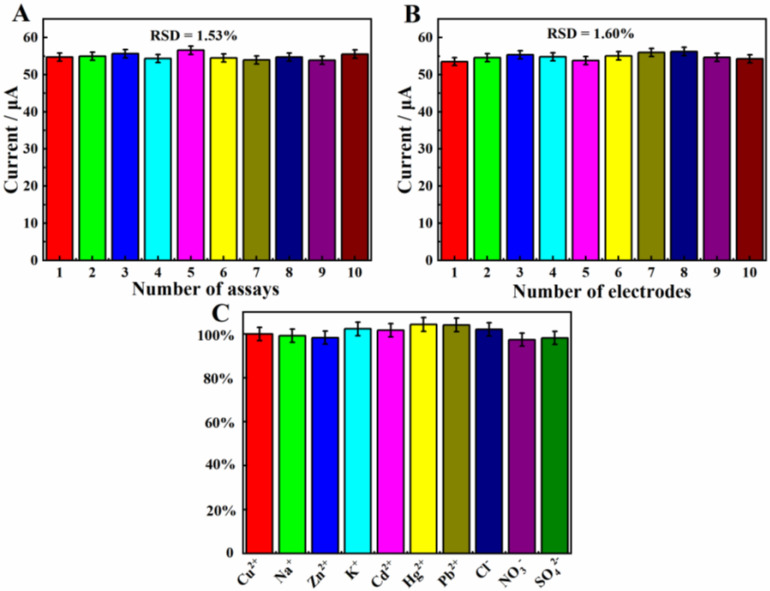
(**A**) Repetitive DPASV responses regarding β-CD/CMK-8/GCE with the same electrode; (**B**) DPASV responses of β-CD/CMK-8/GCE for 10 different electrodes. (**C**) DPASV responses of β-CD/CMK-8/GCE with 0.1 M ABS solution (pH 5.0) containing Cu^2+^ (1 mg·L^−1^) in the presence and absence of 100-fold common ions (Cu^2+^, Na^+^, Zn^2+^, K^+^, Cd^2+^, Hg^2+^, Pb^2+^, Cl^−^, NO_3_^−^, SO_4_^2−^).

The repeatability of β-CD/CMK-8/GCE toward 1.0 mg·L^−1^ Cu^2+^ was explored by using one modified electrode (Figure 8A). After 10 consecutive measurements, there was no significant loss between these electrochemical signals, and the relative standard deviation (RSD) was calculated to be 1.53%. The significant difference between the 10 sets of date was estimated by using the t-test method. The *p*-value was calculated as 0.063 using SPSS 18.0 software. Generally, the level of statistical significance was set at 0.05 (α = 0.05). As the calculated *p*-value was bigger than 0.05, there was no significant difference between the 10 sets of current data. This result demonstrates that the repeatability of β-CD/CMK-8/GCE was satisfactory.

The reproducibility of the β-CD/CMK-8/GCE was confirmed by detecting 1.0 mg·L^−1^ Cu^2+^ with 10 individual modified electrodes. As shown in Figure 8B, the response of peak current remained almost stable and the relative standard deviation (RSD) was calculated to be 1.60%. Similarly, the result of *p*-value was calculated to be 0.052, using SPSS 18.0 software, which was bigger than our considered significance level 0.05, indicating that there was no significant difference between the measured currents. The result showed that β-CD/CMK-8/GCE owns excellent reproducibility.

Selectivity is also an important factor in evaluating the performance of a sensor, which could be further evaluated by selectivity factor (SF). Here, SF = I / I_0_ · 100%. I and I_0_ stand for the DPASV responses of β-CD/CMK-8/GCE toward 1.0 mg·L-1 Cu^2+^ under 100-fold common ions (Cu^2+^, Na^+^, Zn^2+^, K^+^, Cd^2+^, Hg^2+^, Pb^2+^, Cl^−^, NO_3_^−^, SO_4_^2−^) and 1.0 mg·L^−1^ Cu^2+^, respectively. As shown in Figure 8C, the SF values were calculated as 99.2%, 98.3%, 102.3%, 101.7%, 104.3%, 104.1%, 102.1%, 97.4%, 98.2%, respectively. These results revealed that the common ions had no effect on the detection of Cu^2+^, which indicated the excellent selectivity of β-CD/CMK-8/GCE toward Cu^2+^.

### 2.9. Real Sample Analysis

Tap water was used as the real sample to verify the practicability of β-CD/CMK-8/GCE in the analysis of real samples. The tap water sample was firstly filtrated with 0.45 μm filter, and the pH value was adjusted to 5.0. Subsequently, different concentrations of Cu^2+^ standard solutions were spiked separately into the tap water sample and tested using β-CD/CMK-8/GCE. The data are summarized in Table 2. As shown, the recovery of Cu^2+^ was from 99.38% to 104.0%, and the RSD value was less than 4%, indicating that the β-CD/CMK-8 /GCE was feasible for the detection of Cu^2+^ in the tap water sample.

## 3. Experimental Section

### 3.1. Materials

Mesoporous carbon (CMK-8) was obtained from XF Nano Co., LTD (Nanjing, China). β-cyclodextrin (β-CD), N, N-Dimethylformamide (DMF), KOH, HCl, HNO_3_, H_2_SO_4_, NaOH, Cu(NO_3_)_2_·3H_2_O, and Cd(NO_3_)_2_·4H_2_O were supplied from Aladdin (Shanghai, China). Absolute ethanol was obtained from Yishi Chemical Co., Ltd. (Shanghai, China). The water used in this work was deionized water.

### 3.2. Instruments

Scanning electron microscopy (SEM) (Hitachi Company, Tokyo, Japan) and X-ray diffraction (XRD) (Rigaku Corporation, Tokyo, Japan) were used for the material characterization. All electrochemical measurements were carried out in CHI 660E electrochemical workstation (CH Instrument Co., Ltd., Shanghai, China). A conventional three-electrode cell, consisting of a working electrode (bare or modified glassy carbon electrode (GCE)), a opposite electrode (platinum electrode) and a reference electrode (saturated calomel electrode), was employed in this work.

### 3.3. Preparation of the β-CD/CMK-8 Composite

The amounts of 6 mg β-CD and 3 mg CMK-8 were added to 3 mL distilled water and 3 mL DMF, respectively, which were then ultra-sounded for 30 min to obtain uniform dispersion. Finally, β-CD/CMK-8 was obtained by mixing 1 mL β-CD dispersion and 1 mL CMK-8 dispersion uniformly.

### 3.4. Fabrication of β-CD/CMK-8/GCE

**Scheme 1:**
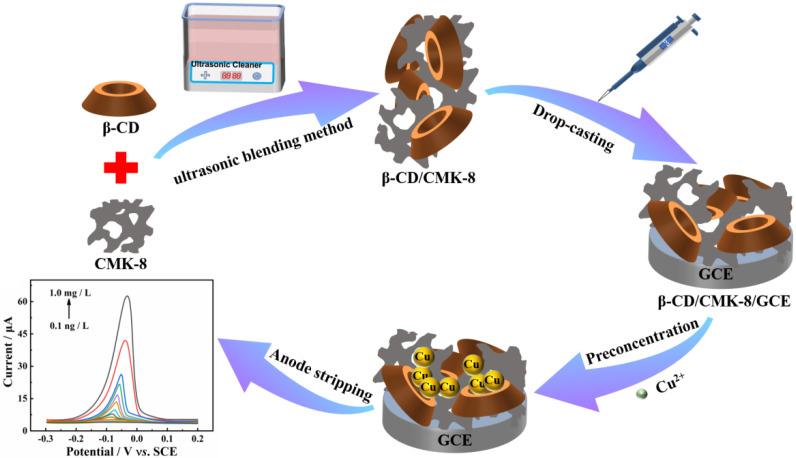
Schematic illustration of the preparation process of β-CD/CMK-8/GCE and the sensing strategy for Cu^2+^.

GCE was polished with 0.05 μm alumina powder, and washed with ethanol and deionized water successively. Subsequently, 3 μL β-CD/CMK-8 suspension was dropped on bare GCE by pipette gun and dried under the electric blast drying oven. The synthetic route of β-CD/CMK-8/GCE and sensing strategy of the Cu^2+^ sensor are shown in Scheme 1.

## 4. Conclusions

In this work, an effective electrochemical sensor for Cu^2+^ was developed, based on the synergistic effect of β-CD and CMK-8. The multiple adsorption sites of β-CD and the good conductivity of CMK-8 enabled β-CD/CMK-8 to exhibit excellent detection performance with a satisfactory detection limit of 0.3 ng·L^−1^ and linear range from 0.1 ng·L^−1^ to 1.0 mg·L^−1^. At the same time, the repeatability, reproducibility and selectivity of β-CD/CMK-8 /GCE toward Cu^2+^ were satisfactory. In addition, the novel sensing platform proved useful to detect Cu^2+^ in tap water, demonstrating good application prospects.

## Figures and Tables

**Table 1:** Comparison of the detection performance in various reported electrochemical Cu^2+^ sensors.

Electrode Substrate	Measurement Technique	Linear Range (μg·L^−^^1^)	LOD (μg·L^−^^1^)	References
AuNPs-GR ^a^/GCE	ASV	0.32–6.4	0.0018	[34]
SSA/MoS_2_/o-MWCNTs ^b^/GCE	DPASV	6.4–704	3.648	[35]
Trp-RGO ^c^/GCE	DPASV	15.36–3072	4.096	[36]
C-Dot-TPEA ^d^/GCE	DPASV	64–3840	6.4	[37]
[PAH-GO ^e^]n/GCE	DPASV	32–3200	22.4	[38]
β-CD/CMK-8/GCE	DPASV	0.001–1000	0.0003	This work

Note: ^a^: graphene and AuNPs; ^b^: oxidized multi-walled carbon nanotubes functionalized with 5-sulfosalicylic acid/MoS_2_ nano-sheets nanocomposites; ^c^: Tryptophan non-covalent modification of reduced graphene oxide; ^d^: Carbon Dot-TPEA Hybridized; ^e^: Layered graphene nanostructures functionalized with NH_2_-rich polyelectrolytes.

**Table 2:** Recoveries of trace Cu^2+^ in tap water sample (*n* = 3).

Sample	Added (μg·L^−^^1^)	Founded (μg·L^−^^1^)	Recovery (%)	RSD (%)
1	0	-	-	-
2	0.5	0.52 ± 0.02	104.0	3.85
3	5.0	4.98 ± 0.09	99.63	1.81
4	50.0	49.69 ± 0.79	99.38	1.59
5	100.0	101.0 ± 2.31	101.0	2.29

## Data Availability

The data presented in this study are available in article.

## Sample Availability

Samples of the compounds are not available from the authors.

## Abbreviations

Acetic acid	HAc
Apparent dissociation constant	K_D_
Atomic absorption spectrophotometer	AAS
Acetate buffer solution	NaAc-HAc
Cyclic voltammetry	CV
Copper ion	Cu^2+^
Differential pulse anodic stripping voltammetry	DPASV
Electrochemically effective surface area	A_eff_
Electrochemical impedance spectroscopy	EIS
Geometric surface area	A_geom_
Graphite furnace atomic absorption spectroscopy	GF-AAS
Glass carbon electrode	GCE
Heavy metal ions	HMIs
HAc-NaAc buffer solution	ABS
inductively coupled plasma atomic emission spectrometry	ICP-AES
Limit of detection	LOD
Maximum binding capacity	Q_max_
Mesoporous carbon	CMK-8
Multi-walled carbon nanotubes	MWCNTs
Oxidized multi-walled carbon nanotubes	o-MWCNTs
Phosphate buffer solution	PBS
Roughness factor	R_f_
Selectivity factor	SF
Sodium acetate	NaAc
Scanning electron microscopy	SEM
X-ray diffraction	XRD
β-cyclodextrin	β-CD
5-sulfosalicylic acid	SSA
